# Supplementary material for: Are carbon emissions trading and green financial instruments synergistic? -Comprehensive quantitative research based on content analysis
Source: PLoS One. 2024 Mar 7;19(3):e0298601. doi: 10.1371/journal.pone.0298601 (PMC10919601; doi:10.1371/journal.pone.0298601)
Supplement: S1 Data — (ZIP) [file pone.0298601.s001.zip › Data/地级市Z_F工作报告.zip!/╔╜╬≈/╠1⁄2╘¡╩╨/└┤╫╘╣1⁄2╓┌║┼í╛┬φ┐╦╩2╛▌═°í┐_2007.docx]

各位代表：

现在，我代表市政府向大会作报告，请予审议，并请各位政协委员和其他列席人员提出意见。

一、本届政府工作回顾

本届政府自2002年3月市第十一届人民代表大会第一次会议选举产生以来，任期届满。

五年来，在省委、省政府和市委的正确领导下，在市人大和市政协的有力监督支持下，我们以“三个代表”重要思想和科学发展观为指导，团结带领全市人民迎难而上，奋力拼搏，率先发展，顺利完成了国民经济和社会发展第十个五年计划，实现了“十一五”规划的良好开局，推动国民经济和社会发展迈上了新的台阶。

五年来，全市地区生产总值由451.2亿元增加到1013.4亿元，年均增长13.9%；财政总收入由64.2亿元增加到192.2亿元，年均增长24.5%；一般预算收入由24.2亿元增加到75.3亿元，年均增长25.5%；规模以上工业增加值由99.5亿元增加到341.6亿元，年均增长20.6%；全社会固定资产投资累计达到1639.4亿元，年均增长32.5%；社会消费品零售总额由206.7亿元增加到436.5亿元，年均增长16.1%；外贸进出口总额由13.2亿美元增加到41.1亿美元，年均增长25.5%；城镇居民人均可支配收入由6500元增加到11741元，年均增长12.5%；农民人均纯收入由2738元增加到4917元，年均增长12.4%；城镇养老、医疗、失业保险覆盖率分别达到96%、86.5%和97%；高新技术产业增加值占GDP比重达到5.4%；森林覆盖率达到13%，矿区煤炭资源回采率达到52.5%，空气质量二级以上天数由120天增加到261天，净增141天。本届政府提出的主要经济社会发展指标全部完成！

发展成就令人振奋，发展历程令人难忘。五年来，我们深入推进结构调整，不锈钢、富士康等一批重点项目达产达效，第三产业稳步发展，产业结构不断优化；大力开展城市环境六项综合整治，汾河治理美化一期、长风大街、集中供热扩网等重大城市建设工程顺利竣工，城市面貌发生显著变化；深化行政审批制度改革，国企改革实现突破，举办建城2500年庆典，全力搞好招商引资和招才引智，改革开放不断深入；万众一心战胜“非典”疫情，公共事件应急处理能力显著提高，教科文卫等各项社会事业全面推进，经济社会协调发展；大力完善城乡社会保障体系，农村新型合作医疗和低保制度全面实行，治理水土流失，实施农村饮水安全工程，城乡生产生活条件明显改善，改革发展成果惠及更多群众。

五年来，我市先后荣获全国就业和再就业先进城市、全国双拥模范城五连冠、全国社区建设示范城市、全国优秀旅游城市、全国科技进步城市、全国节约用水先进城市、全国万村千乡市场工程先进集体、全国职业教育先进单位、全国文化市场建设先进单位、全国实施体育锻炼标准先进城市、全国无偿献血先进城市、全国无障碍建设先进城市、全国人民防空先进城市、全国法制宣传教育先进城市、全国社会治安综合治理优秀城市、创建全国文明城市工作先进城市和全国民族团结进步模范集体等荣誉称号，汾河景区、城西水系分别荣获迪拜国际改善人居环境最佳范例奖、中国人居环境范例奖，城市的吸引力和影响力进一步增强。

过去的五年，是太原迈向现代化的加速发展期，是太原全面建设小康社会的关键发展期，是太原历史上的最好发展期，谱写了辉煌篇章。这些成绩的取得，是认真贯彻中央和省各项重大决策的结果，是省委、省政府和市委正确领导的结果，是市人大、市政协和各位代表、各位委员监督支持的结果，是全市人民团结奋斗的结果。我代表市政府向辛勤工作在各条战线的工人、农民、知识分子和各级干部，向大力支持政府工作的人大代表、政协委员、各民主党派、工商联、人民团体、无党派人士，向关心帮助太原发展的中央、省驻并单位和驻并部队、武警官兵，向积极参与太原建设的各界朋友，表示崇高的敬意和衷心的感谢！

回顾五年的丰富实践，我们深刻体会到：要把太原的各项工作做好，必须在科学发展观指导下，追求又好又快发展；必须创新发展理念，努力探索具有太原特色的率先发展之路；必须推进经济结构绿色转型，走新型工业化和特色城镇化道路；必须坚持改革开放，为各项事业注入新的生机与活力；必须把广大人民群众的利益放在首位，加快发展社会事业，努力构建和谐社会；必须加快转变政府职能，为经济社会发展营造良好环境。

各位代表，在对五年工作简要回顾的基础上，现就2006年政府工作向大会报告。

2006年，市政府认真贯彻落实中央、省委省政府和市委的一系列决策部署，全力抓好“十一五”规划的开局、布局工作，取得了新的成绩。

（一）壮大发展实力，各大产业提质增效，城市建设高起点展开，又好又快发展格局初步形成

工业经济提质增效。加快构建绿色产业体系，以不锈钢及深加工为龙头的冶金产业、以煤化工为重点的化工产业、以重矿和煤机设备为代表的装备制造产业产值均增长25%以上。积极培育大企业、大集团，“四百工程”项目建设明显见效，新东方铝业、蓝星TDI等100个重点达产达效项目，新增产值78亿元，成为全市经济增长的重要支撑和拉动力量。高新区、经济区、民营区工业总产值分别增长24.3%、43.9%、31.4%。全市规模以上工业总产值完成1076亿元，增长18%；工业经济效益综合指数达到155.6，再创历史新高。

农村经济稳步发展。农村经济总收入455亿元，增长8.2%。农业产业化龙头企业达到180家，实现销售收入47亿元，增长17.5%。全面启动“百村试点、千村美化”工程，完成103个新农村建设试点村规划编制工作。把“康庄生态富民沼气工程”作为新农村建设的龙头，建设沼气池3765座。建设农村公路872公里。荒山造林6万亩。高标准建设绿色节能温室3万间。治理水土流失面积20万亩。解决了9.3万人的饮水困难和水质不达标问题。全面完成娄烦8个村、阳曲7个村整村推进项目。农村科技信息“村村通”达到87.3%，农村教育综合改革经验在全省推广。选派170名机关干部深入农村开展工作。2.7万农村贫困人口脱贫。

内外贸易日益活跃。研究出台加快流通业发展的实施意见和加快现代服务业发展的政策意见。开展“万村千乡市场工程”建设试点，新建、改造乡村“农家店”572家。实施“老字号”振兴工程，老陈醋、六味斋等5家企业被国家认定为“中华老字号”。华宇、美特好、唐久分别进入全国零售业百强、中国服务业500强和全国连锁业500强。物流业发展迅速，被国家列为物流园区建设试点城市。加快粮食安全体系建设，粮食市场保持稳定。实施“食品药品放心工程”，市场秩序日趋规范。旅游业发展迅猛，旅游总收入达到109.6亿元，增长36.2%。出口产品结构明显优化，外贸进出口总额完成41.1亿美元，增长22%。对外劳务合作增长21.8%。

城市建管水平有所提高。引进国际高水平规划设计机构，围绕“三城联动”的整体布局，编制完成近期建设规划、城市综合交通规划等一批详细规划。南部新区详细规划、晋阳新区概念规划、旧城改造规划等重点规划项目加速推进。加强城市基础设施建设，长风东大街、西北环连接线竣工通车，完成体育路和11条小街小巷的拓宽改造。机场大道、滨河东路南延、汾河治理美化二期、河西热源厂、采煤沉陷区综合治理等重点工程进展顺利。天然气入并工程实现供气。建成城市生活垃圾焚烧发电工程，城市生活垃圾无害化处理率达到80%。集中供热扩网740万平方米，集中供热普及率达到80.7%。城中村改造拆迁40.5万平方米。旧城改造和经济适用房开复工129.5万平方米。加强城市管理，强化规划权威，拆除违法建筑27.3万平方米，调查控制违法建设2000余件、342万平方米。清偿拖欠工程款29.1亿元，清偿率99.2%。

（二）转变发展模式，资源节约、环境友好型社会建设有序推进，转型发展开始起步

全面加强资源节约与保护。严格实施“绿色高压线”，否决和整顿145个不符合产业政策的项目，取缔161家不符合产业政策的小企业。认真搞好煤炭资源整合和有偿使用，压减关闭煤矿矿井121座。关闭小机焦项目9个，压缩产能118万吨。制定了工业能耗指标和重点行业、重点企业产品单耗指标，90%的工业企业建立了节能管理制度。加强对能耗5000吨标准煤以上单位的监督检查，对能效不达标的设备实施改造。开展建筑节能检查，整改建设项目39个。在宾馆、饭店、学校等10个非工业领域开展节能、节水示范试点，万元地区生产总值水耗下降10.5%。

努力提高资源综合利用水平。编制完成清洁生产规划，实施重点清洁生产项目12项。重点组织开展化工、冶金等传统产业的循环经济试点工作，实施重点调产和技改项目450项，建成投产192项，提高了用能效率。按照“同业入园、形成循环”的思路，加快工业园生态化改造，经济区废弃物基本实现循环利用。扶持太钢粉煤灰综合利用等一批重点项目，工业固体废弃物处置利用率达到93.7%。

大力加强环境治理和保护。深入推进“蓝天碧水”工程，实施8项环保创建示范工程，开展控制结构性污染、削减污染物排放总量、调整燃料结构、遏制扬尘污染四项整治行动。实施19项重点污染治理工程，万元GDP污染物排放量下降 20.2%。拆除燃煤锅炉496台，新建高污染燃料禁燃区6平方公里。加强城市生态建设，新增绿化面积216.9公顷，绿化覆盖率提高1.2个百分点，人均绿地面积达到8.38平方米。

（三）强化发展动力，体制机制、科学技术和发展环境创新取得进展，创新发展加快推进

创新体制机制，各项改革不断深化。国有资产监管体制初步理顺，国企改革路径初步打通。充分利用并轨政策，2.4万名职工实现人资分离和劳动关系调整，为资产运营奠定了良好基础。按照“多方筹资、整体打包、回购债权、清偿债务”的思路，回购不良债权20.7亿元，使企业2000多亩土地解封，100余宗法律官司解除，40多个担保企业解脱。以改制为手段，以园区为载体，积极进行产业重构与整合，推进“腾笼换鸟”。全面推进商行改革，完善法人治理结构，加强风险处置，不良资产清收3.1亿元，置换25亿元，全市金融生态环境得到改善。加快文化体制改革，成立了文广局、文广集团。深化财政体制改革，优化支出结构，各项重点支出得到较好保障。城市管理体制改革、医疗卫生体制改革、价格管理体制改革扎实推进，体制活力进一步显现。

加快科技创新，发展后劲持续增强。研究与开发经费占GDP比重达到1.05%。安排科技项目202项，启动科技创新“双十工程”，确定10家首批创新型试点企业和10个重大自主创新项目，在项目、资金、政策等方面给予重点扶持。实施“技术创新引导工程”，开展创新型企业试点、产学研技术联盟试点。科技企业孵化面积新增2.1万平方米，申报专利1458件，增长31.6%。每10万人专利申请数42项，科技成果转化率32%。科技进步对GDP增长的贡献率达到53.8%。清洁能源、制造业信息化和专利工作三项试点通过国家验收。高新区4项科技项目列入国家火炬计划，煤化工技术研发基地正式成立。

开展“环境创新年”活动，发展环境进一步优化。围绕“创优政务环境、整治城市卫生、创建文明交通”三个重点，努力打造环境优美、服务高效的城市新形象。进一步清理行政许可、行政审批和各类服务性事项，取消130项，减幅27%；对8个部门的121项收费按下限收取，降幅50%；对54个部门的年检、年审、换证事项进行清理，取消36项，减幅37%。开展投资促进服务，全面推行全程代办制、服务承诺制、首办负责制、限时办结制、超时默认制和挂牌保护制。推进机关行政效能建设，开展公务员“百岗评议”活动，及时纠正和处理损害政务环境的行为。设立“12345”政府便民服务热线，搭建政府与市民交流平台。深入开展城市环境卫生整治活动，清理非法储煤场存煤330万吨，清理垃圾84万吨，取缔无证摊点和占道经营6.5万处。严厉查处各类交通违法行为，交通事故起数、死亡人数分别下降2.8%和1.9%，直接经济损失下降30.4%。

（四）优化发展格局，招商引资实现突破，民营经济发展迅速，创新转型活力加速释放

招商引资取得丰硕成果。建立招商引资项目库，制定落实优化外商投资环境的11条措施和鼓励外商投资的14条优惠政策。组团参加中博会、港洽会、沪洽会等各类经贸洽谈活动，签约项目147个，引进资金74.5亿美元，项目数量和引资额度创历史新高。狠抓签约项目的履约工作，新批外商投资企业51户，增长45.7%；项目总投资10.6亿美元，增长117.2%。合同利用外资4.9亿美元，增长100.9%；实际到位外资7.25亿美元，占全省的53.8%。

民营经济发展强劲。加大对民营经济扶持力度，通过财政贴息、银企对接等途径，解决融资难题。民营经济增加值完成456亿元，增长22.3%；上缴税金78.9亿元，增长23.4%。乡镇企业增加值完成176亿元，增长18.9%。全市规模以上民营企业达到945家。民营经济占地区生产总值的比重达到45%。

（五）丰富发展内涵，经济发展与社会事业、人民共富同步推进，和谐社会建设逐步加强

承诺为群众办的实事全部完成。认真解决上学难问题，免除19万名农村义务教育阶段学生和城市贫困学生的学杂费，为4万名贫困生免费提供教科书，为9526名寄宿生提供生活补助。建设城镇学校标准操场115个。建成47所高标准九年一贯制学校和寄宿制小学。着力缓解看病难问题，设立农村巡回医院送医下乡，在全市113所医院开设惠民济困门诊。10县区全部实施新型农村合作医疗，参合率84.4%。抓紧解决住房难问题，安康居住工程竣工93.3万平方米。矿工棚户区改造竣工9.4万平方米。采煤沉陷区治理开工53.7万平方米。维修加固城乡住房44.6万平方米。切实解决行路难问题，新改建村村通水泥路553公里，完成通达通畅工程281.5公里。行政村通客车率达到97%。大力解决就业和社会保障难的问题，新增城镇就业岗位14万个，下岗再就业5.8万人，安置“4050”等就业困难人员9252人。为22.6万名退休人员补发养老金4.3亿元。

努力解决群众关心的民生问题。全面完成下岗职工基本生活保障向失业保险并轨，出中心并轨3.1万人。安置3539名零就业家庭成员上岗。城镇登记失业率为3.6%。为下岗失业人员提供小额担保贷款2506万元。提前两年全部免征农业税及附加。占人口20%的低收入者人均收入城镇增长7.4%、农村增长13%。居民消费价格总水平涨幅为1.6%。征缴社会保险费33亿元，创历史新高。提高低保标准，为8.1万城市低保对象、5.3万农村低保对象发放低保金1.4亿元，实现了应保尽保。完善城乡社会救助体系，为困难群众发放救济款740万元。狠抓安全生产，亿元地区生产总值生产安全事故死亡人数下降22.5%，该降低的各项主要指标继续保持全省最低。认真做好信访和人民调解工作，实行重点信访案件领导“包案”制度，妥善处理了一批多年累积的信访问题，赴省集体上访批次、人次下降13%和15.5%。严密防范和严厉打击境内外敌对势力和敌对分子的渗透破坏活动，积极化解社会矛盾纠纷，预防和处置群体性事件，确保省城政治稳定。加强社会治安综合治理，有力维护了社会稳定。

积极发展社会事业。启动“百校兴学”工程，全面推进中小学义务教育标准化建设，小店、清徐通过省验收。分离63所中央、省属企业学校。大力普及学前教育，入园率达到90%。高中阶段毛入学率达到86%。创建2所国家重点职业学校。改造乡镇卫生院、农村卫生所169所，县乡村三级医疗卫生机构达标率44.8%。抓好计划生育工作，人口出生率、自然增长率分别为8.05‰和4.12‰。以“践行八荣八耻，建设创新型城市”为主题，开展形式多样的文化活动。实施“文化村村通”工程，丰富群众文化生活。成功承办第12届省运会，金牌、总分均居全省第一。人均公共体育场馆面积达到0.91平方米，小轮车训练基地被列为国家级基地，获得小轮车世锦赛暨奥运预赛主办权。深入开展精神文明创建活动，城乡文明程度进一步提高。

国防后备力量建设取得新成绩。民兵和预备役部队建设成效显著，国防动员一体化建设扎实推进，较好完成了执勤保障、抢险救灾、维护稳定等任务。国防教育有效开展，双拥建设在“五连冠”的基础上又有新的提高。外事、侨务、气象、防震、档案、文物、地方志、民族、宗教、广电、新闻出版、人民防空等工作取得新成绩，老龄、妇女、残疾人、关心下一代等事业都有新进步。

各位代表，在肯定成绩的同时，还必须清醒地看到，我市经济社会发展还面临着很多制约和挑战，与建设新太原的要求相比，还有不小的差距：资源型、高消耗产业仍占主要地位，可持续发展的能力不强；城市建设、管理水平不高，承载能力与省会城市地位不相适应；生态环境治理和保护形势严峻，污染严重、生态脆弱的问题比较突出；科技创新水平比较低，自主创新能力还不够强；影响和谐社会建设的困难和问题比较多，农业基础设施滞后，农民增收困难，城乡居民就业、医疗、教育和社会保障等问题还没有根本解决，信访总量居高不下，安全生产形势不容乐观；体制机制创新滞后，影响发展的深层次障碍仍然较多；环境创新任务繁重，政府职能转变需要进一步加快。对于这些问题和薄弱环节，我们一定要高度重视，采取有力措施，切实加以解决。

二、对下届政府工作的建议

未来五年，新一届政府肩负着建设创新型城市、推进经济社会绿色转型，圆满完成“十一五”规划，建设服务全省、影响全国、吸引世界新太原的历史重任。在前进的道路上，我们面临着一些更为有利的发展机遇。从发展环境看，省委、省政府在结构调整、城市建设等方面加大对太原的支持力度，国家细化和落实促进中部崛起的政策措施，我们将获得更多的支持。从发展质量看，我市一批重点监控项目陆续投产达效，对全市经济的拉动和支撑作用不断增强，为又好又快发展奠定了坚实基础；固定资产投资结构明显优化，投入农村、科技、环保建设的资金持续增加，经济增长与财政增收、人民致富之间的协调性增强。从发展态势看，全市GDP近年来一直保持两位数增长，显现出平稳发展的态势；人均GDP已达到3782美元，接近中等发达城市水平。只要我们坚定信心，抓住机遇，群策群力，乘势而上，就一定能够推动经济社会发展迈上新的台阶。

新一届政府工作的指导思想是：高举邓小平理论和“三个代表”重要思想伟大旗帜，全面落实科学发展观和省委、省政府走出“四条路子”、实现“三个跨越”的重大决策，坚决贯彻创新型城市建设主战略，以创新发展模式、推进绿色转型为战略着力点，围绕新型工业基地和特色文化名城总体目标，加速构建资源节约、环境友好的绿色经济体系，加速构建生态宜居、协调共进的新型城乡体系，加速构建全面发展、共同富裕的和谐社会体系，加速构建延续文脉、科学先进的特色文化体系，加速构建充满活力、适应科学发展的新型体制机制，努力建设服务全省、影响全国、吸引世界的新太原。

按照这一指导思想，新一届政府要全力实现以下奋斗目标：

——全市地区生产总值年均增长12%，人均地区生产总值年均增长10.5%，规模以上工业增加值年均增长13%，财政总收入年均增长14%，到2011年分别达到1785亿元、6230美元、630亿元和370亿元，经济发展质量效益大幅提升，实力更强。

——固定资产投资年均增长15%，城市规划建设管理水平迈上新台阶，南部新区重点工程成为太原21世纪新标志，晋阳古城文化旅游价值全面彰显，老城区宜居程度大幅提升，城市更美。

——农民人均纯收入年均增长11%，到2011年达到8285元，新农村建设任务加快推进，生产生活条件明显改善，农村更富。

——万元地区生产总值能耗年均下降6.4%，五年下降28%；万元地区生产总值二氧化硫、化学需氧量年均下降10%，五年下降41%，发展模式加快转变，生态建设取得实效，环境更好。

——招商引资规模不断扩大，社会消费品零售总额、地区外贸进出口总额年均增长15%和14%；国企改革任务全面完成，各项改革继续深化，动力更足。

——城镇年均新增就业岗位14万个，城镇登记失业率每年控制在4%以内，人口自然增长率每年控制在4.5‰以内，城镇居民人均可支配收入年均增长10%，到2011年达到1.89万元，各项社会事业全面推进，民主法制和精神文明建设水平明显提升，社会更和谐。

要实现上述目标，新一届政府应紧紧围绕又好又快发展主题，把握率先科学发展要求，扭住整体创新、绿色转型这条主线，全力推进“四个发展”：

一是全力推进创新发展。全面贯彻创新型城市建设主战略，大力推进思想观念创新，用创新的理念破解难题，用创新的精神开创未来；推进科学技术创新，深入实施人才强市战略，提升区域技术研发中心的地位，增强优势产业和特色领域的自主创新能力，力争建成全国科技进步示范市；推进体制机制创新，进一步深化改革，扩大开放，为科学发展提供体制机制性保障，让各类要素的潜力竞相迸发；推进发展环境创新，以环境的优化提升城市竞争力，以环境的创新赢得发展新机遇，以环境的改善提高人民生活质量；推进文化氛围创新，大力倡导敢于探索、勤于创造、勇于竞争的创新文化，使创新成为全市人民的自觉行动。

二是全力推进绿色发展。在科学发展观指导下，整体推进发展模式的绿色转型。构建绿色产业体系，发展循环经济，推进节能降耗，综合开发、榨干炼净、高效利用自然资源，培育和发展标志性名牌绿色产品、绿色企业、绿色产业和绿色园区，打响“太原制造”的绿色品牌；加快发展绿色农业，建设富有特色的绿色有机经济区，促进农村生产生活方式的绿色转型和根本变革，建设绿色、生态、富裕、和谐的社会主义新农村；加快推进绿色服务业发展，推广绿色行业标准，努力建成具有现代化、知识化、生态化特征的绿色服务业新框架、新体系；拉开绿色城市化框架，打造亮点片区，彰显文化品位，改善人居环境，加快建设资源节约型和环境友好型社会。

三是全力推进和谐发展。把构建社会主义和谐社会摆在突出的战略位置，更加注重支持“三农”，稳步缩小城乡差距，促进城乡协调发展；更加注重发展社会事业，完善基础保障，强化社会管理和公共服务的职能，大力改善城乡居民教育、文化、卫生、交通、居住条件，满足人的全面发展的需要；更加注重以人为本，在改革发展中认真解决群众关心的突出问题，坚决维护群众的切身利益；更加注重创造公平公正的社会环境，妥善处理不同利益群体关系，千方百计扩大就业，健全社会保障体系，关注帮助困难群体；更加注重公共安全，强化市场监管，狠抓安全生产，建设平安省城；更加注重缩小收入差距，坚持共同富裕，使广大人民群众共享改革发展的成果。

四是全力推进跨越发展。追求卓越，争先创优，各项经济社会发展指标，该高的要高于全国省会城市平均水平，该低的要低于全国省会城市平均水平，好中求快，做大总量，早日跻身全国先进省会城市的行列。进一步强化省会意识，奋力在结构调整、城市建设、构建和谐、改革开放等方面实现全方位率先发展，发挥好龙头带动作用；加强区域交流合作，加快建设太原经济圈，形成基础设施完善便捷、资源优化配置、市场健全畅通、产业布局合理的区域经济一体化发展格局，成为对全省具有强大带动作用的新型经济增长极。加快建设不锈钢生产、铝镁合金加工、新型装备制造业“三大基地”和区域性现代商务、物流、旅游、金融“四个中心”，高起点跨越式发展文化产业和事业，使太原成为国家区域发展战略的重要支撑和闻名世界的历史文化名城。

根据以上指导思想、奋斗目标和战略重点，在去年开局起步的基础上，新一届政府应分三步推进整体创新与绿色转型。一是重点突破，用1到2年时间，突破结构调整、城市建设、国企改革、“三农”发展等经济社会发展的重点与难点，打破发展道路上的“瓶颈”制约，构建整体创新与绿色转型的基本格局；二是整体推进，用2年左右时间，在经济社会各领域推进全面创新和绿色转型，创新转型初见成效；三是巩固提升，用1年左右时间，巩固扩大创新转型成果，主要绿色考核指标达到或超过全国省会城市平均水平，为新太原建设奠定坚实基础。

各位代表，2007年是完成新一届政府战略任务的首战之年，也是“十一五”规划全面展开、重点突破的关键之年。政府工作要以科学发展观为指导，认真贯彻省、市九次党代会精神和建设创新型城市主战略，奋力在事关全局的战略重点上实现六个新突破：加快经济结构调整，在推进发展模式绿色转型上取得新突破；搞好“三城”建设，在提升城市形象上取得新突破；积极发展现代农业，在新农村建设上取得新突破；加快改革开放和自主创新，在体制机制创新上取得新突破；优化发展环境，在提高行政效能上取得新突破；更加关注民生，在构建和谐社会上取得新突破。

按照上述要求，围绕全面完成省政府44项考核指标，着重做好以下八个方面的工作：

（一）强化绿色理念，制定绿色标准，全方位、宽领域、深层次推进绿色转型

着眼于又好又快发展，立足于资源环境的承载力，研究制定、逐步推出和全力实施切实可行、科学合理、覆盖各行各业的绿色标准体系以及绿色考核指标体系，在经济社会各领域、生产生活各方面全力推进绿色转型，奋力建设资源节约型和环境友好型城市。

大力发展循环经济。完成全市循环经济规划和工业循环经济发展规划编制，推广太钢、太化、煤气化、狮头水泥等试点经验，重点开展冶金、化工等传统产业试点，抓好屯兰、马兰等煤矿瓦斯综合利用工程和太钢干熄焦工程等示范项目。全面完成煤炭、电力、焦化、水泥、化工、电镀等行业25个企业的强制性清洁生产审核，鼓励和引导非强制性企业主动开展清洁生产审核，实现节能降耗，减排增效。高新区、经济区、民营区、不锈钢园区要积极创建生态工业园区。推动清徐和古交创建循环经济示范县（市），发展县域循环经济。

强力推进节约型社会建设。建立资源节约责任制，把资源节约责任纳入各项工作岗位职责，纳入各部门日常管理和工作考核。狠抓重点行业和企业节能降耗，大力推广先进适用的节能新技术、新工艺、新设备和新材料，在年耗能5000吨标准煤以上企业中开展“百家企业节能行动”，规模以上工业企业增加值能耗下降7.8%。积极发展太阳能等替代能源。加快制定实施“兴水战略”，加强节水工作，促进水资源的合理开发利用和有效保护。加强建筑节能工作，新建建筑严格实施节能50%的设计标准，新建民用建筑实施供水“一户一表”和供热“分户控制、分户计量”。落实国家土地资源保护政策，严格国土资源管理。加强矿产资源综合开发利用、中水等再生资源回收利用和生活垃圾资源化利用，不断提高资源利用水平。

重点突破自主创新的制约瓶颈。借鉴和推广太钢自主创新的成功经验，开展“技术创新引导年”活动。实施技术创新“双十工程”，重点支持10个创新型试点企业和10个重大自主创新项目；开展产学研战略联盟试点，重点支持10个产学研合作项目。申报省技术创新项目80项，认定5—8户市级企业技术中心，力争2—3户进入省级企业技术中心行列。加快技术研发公共平台、成果转化服务体系和“一网两库”建设，建立10个具有区域特色的产业集群技术联盟试点，引导入园企业全面开展技术合作，提高中小企业自主创新能力。研究与开发经费占GDP的比重达到1.25%，科技成果转化率达到35%以上，科技对全市经济增长的贡献率提高2个百分点。

坚持不懈开展环境治理。建立完善“绿色高压线”，提高建设项目准入门槛，对不符合科学发展观和国家产业政策的新建项目坚决否决，对小冶炼、小钢铁、小焦炭、小化工、小水泥等落后生产能力和设施坚决取缔，对布局不合理的污染严重企业坚决搬迁改造。大力调整燃料结构，抓好集中供热、煤改气工程和清洁燃料的推广使用，新增集中供热面积1000万平方米以上，置换10万户居民生活用煤气，新建9平方公里高污染燃料禁燃区。加大工业企业污染治理力度，严厉打击不法排污企业，严格控制污染物排放总量，完成一电厂烟气脱硫等28项重点治理工程，削减烟粉尘3000吨、二氧化硫2.5万吨。加强地表饮用水源地保护，整治汾河上游引黄导流明渠，支持娄烦县建设“全省生态环保第一县”。加大扬尘污染和机动车尾气污染防治力度，全面完成市区周边非法储煤场的清理整顿工作。

加大生态建设力度。深入开展创建国家园林城市活动，力争完成城西水系二期、唐槐公园南园、学府公园、漪汾公园、植物园建设和南寨公园、玉门河公园续建任务。扩大新动物园、龙潭公园、五一广场景观规划范围，形成规模景区。在旧城区开展园林化单位和社区创建活动，拆墙出绿，见缝插绿，拆旧造绿；在主要出口路两侧高密度、大规模植树造林，重点抓好滨河东西路南北延伸段景观林带、机场大道、迎泽大街等道路的绿化建设。新增绿化覆盖面积200公顷，城市绿化覆盖率和绿地率分别提高1个百分点，人均绿地面积增加0.5平方米。抓好天然林保护、退耕还林、三北防护林、环城林带、交通沿线荒山绿化等生态工程建设，造林总面积25.7万亩，全市森林覆盖率提高1个百分点。

围绕上述各项重点工作，今年要在全市大力开展“绿色十佳”创建活动，在各领域分别评选绿色“十佳村镇”、“十佳企业”、“十佳学校”、“十佳社区”、“十佳医院”、“十佳建筑”、“十佳商店”、“十佳饭店”等先进单位，实现绿色转型的典型引路、重点突破。

（二）继续深化结构调整，加快构建具有省会城市特色的绿色产业体系

按照知识化、清洁化、循环型、低消耗、可持续的要求，改造提升传统产业，加速发展新型产业，努力实现经济、社会、生态相协调和整体效益的最大化。

推动工业经济又好又快发展。围绕新型工业基地建设，继续开展“工业项目推进年”活动，在“四百工程”项目明显见效的基础上，实施“三百工程”，投产、续建、新建各100个项目，对工业经济增长和效益提高的贡献率达到60%。突出抓好太钢150万吨不锈钢冷轧工程、太重煤机成套设备等20个传统产业升级改造项目。重点抓好双喜子午线轮胎一期等10个高新技术项目，提升高新技术和新兴产业在全市经济总量中的比重。全力推进重型汽车、石油钻具和新凯纺织项目，打造制造业新亮点。抓好列入“三个方阵”的22户企业“十一五”规划，对我市确定的30户大企业、大集团完善扶持鼓励政策。全市规模以上工业企业销售收入和上缴利税分别增长10%，经济效益综合指数达到160以上。

积极发展现代服务业。制定和完善促进扩大消费的政策措施，引导企业不断拓展商品和服务范围，满足即期消费，激活潜在消费，创造未来消费。改造提升商贸、餐饮、酒店等传统服务业，实行大型商业网点建设听证制度，搞好社区商业网络建设，加快现代物业管理业发展。加快推广连锁经营、物流配送、电子商务等经营方式，推进山西国际会展中心、中国（太原）煤炭交易市场、中钢集团太原物流园区、武宿公路货运物流园区建设。加快构建适合旅游产业发展的管理体制，集中治理旅游环境，加强旅游产品开发，旅游总收入达到121亿元。

大力提升园区经济。加快民营区搬迁步伐，一手抓阳曲工业新区基础设施建设，一手抓招商引资，提前储备一批项目。加快不锈钢园区二期、三期工程建设，尽快形成50万吨不锈钢深加工能力。加快铝镁工业园建设，整合煤、电等资源，形成煤—电—铝—铝材加工的完整产业链。清徐经济开发区要重点发展煤化工、煤层气综合利用等项目。服装工业园、机械制造园等专业工业园区要抓紧完成规划论证、土地征用等前期工作，启动基础设施建设，使企业尽快入驻。推进“龙头企业特色县”建设，各县区要力争规划建设1—2个绿色工业园区或商贸园区，培育1—3个支撑引领县域经济发展的龙头企业。

清除政策性、体制性障碍，鼓励、支持和引导非公有制经济加快发展，做大非公有制经济总量，非公有制经济增加值增长15%，上缴税金增长10%。

（三）加大对“三农”的支持力度，积极推进新农村建设

加快建立工业反哺农业、城市带动农村的体制机制和政策措施，开辟农业增效、农民增收的有效途径，不断改善农村生产生活条件和整体面貌。

加快发展现代农业。大力发展以无公害食品、绿色食品和有机食品为主的生态农业产业，建设与资源、环境承载力相适应的绿色农业产业体系。农村经济总收入达到505亿元，增长11%。围绕绿色农产品加工和流通中心城市建设，优化农产品加工业整体布局，以大运路为中轴线，规划建设南部水塔老陈醋食品工业园、北部旭美食品工业园和中部市级农产品加工园，扩大农产品加工转化能力，农业产业化销售收入达到50亿元。乡镇企业总产值和增加值均增长15%。

加快农村生态家园和基础设施建设。完善新农村建设规划编制。大力发展农村沼气，突出抓好清徐、娄烦、阳曲3个沼气工程重点县。全面实施“一池四改”，大力推广以沼气工程为主线的康庄生态循环经济。加快农村路网改造，完成县乡公路改造工程457.2公里，通达通畅工程270公里。支持省交通厅开工建设太原—古交—娄烦高速路，做好征地、拆迁等前期工作。加强农田水利基本建设，高标准实施4个农业节水园区和小型水利建设配套及精品小流域工程，新增节水面积1.5万亩。加强农业信息资源整合，提高农村信息化服务水平。大力推进新农村建设试点村社会事业“五个一”工程：每个试点村建1个文化科技活动室（院）、1个卫生所（配备1名具有中专或同等水平的医生）、1个便民连锁店、1处农民休闲健身场所，每个中心村建1所标准化小学。

加快农村服务业发展和脱贫致富进程。着力发展农村第三产业，培育农村商贸、服务、运输、旅游产业。继续推进“关爱老区、帮扶百村”扶贫活动，组织市直机关单位，协调太钢、太铁、西山等大中型企业，加大对贫困老区村的帮扶力度。抓住“两区”开发机遇，实施13个开发项目，加快娄烦、阳曲两县脱贫步伐。

（四）围绕“三城联动”总体布局，大力改善城市人居环境

加大城市规划、建设、管理力度，努力克服传统城市化与人民群众日益增长的人性化、知识化、生态化需求之间的矛盾，力争使城市建设实现跨越式发展。

高标准搞好城市规划。按照“世界眼光、国际水准、时代精神、太原特色”的总体要求，做好2006—2020总体规划修编、报批的前期准备和城市近期建设规划的滚动修订工作，高质量完成南部、东部、北部、西山等重点发展地区的控制性详细规划。建立严格的工作制度和约束机制，对规划区范围内的建设用地和建设活动实行统一、严格的规划监督管理，对不符合城市规划的项目不得批准立项，未编制控制性详细规划和近期建设规划的地块不得出让、转让和开发建设，切实增强执行规划的刚性约束，坚决维护规划的严肃性和权威性。

高质量推进城市建设项目。按照“南移西进、北展东扩、提升品质、重塑形象”的城市建设思路，开工建设50个城市建设项目，总投资137亿元。包括抓好机场大道等城市道桥工程建设项目，启动长风文化商务区等片区建设项目，推进城南污水处理厂一期、侯村垃圾处理场等城市配套建设项目。这是我市城建史上又一次大规模建设活动，是推进绿色城市化、建设新太原的重大战役，各方关注，影响深远。务必本着为人民负责、为历史负责、为未来负责的精神，把这些工程打造成样板工程、精品工程和“阳光工程”，切实把好事办好，取得全胜。要自觉接受人大、政协监督，自觉接受社会舆论监督，纪检、监察、财政、审计等部门提前介入，形成全方位监督机制，进行全程、动态监督，力保项目建设健康进行、优质完工。尤其要处理好建设与便民的关系，加强施工过程管理，强调文明、人本、环保施工，把给人民群众带来的出行和生活不便降到最低程度。

有效加强城市绿色管理。转变城市建设和治理模式，按照绿色标准推进旧城改造、新城建设和城市管理。开展城市环境综合整治，强化细节管理，集中开展“角落问绿”活动，加强对城市出入口、城乡结合部、城中村、小街小巷、居民社区的整治，城中村改造和垃圾清理取得实质性突破。加强公用事业管理，推行园林绿化、市政养护等行业绩效考核，降低养管成本，提高养护资金使用效率。进一步完善12319网络化管理，加快城市管理数字化进程。按照“公交优先”的原则，提升城市公交系统建设管理水平，着力解决“出行难”问题。围绕国际会展中心等省六项重点工程和中国（太原）国际煤炭与能源新产业博览会（煤博会）的召开，全力搞好配套服务工作。配合支持石太高速客运专线和太中银铁路建设。

（五）深化改革和扩大开放，增强经济社会发展的内在活力

围绕绿色转型，大力创新体制机制，以深化改革增动力，以扩大开放聚能量，以优化环境做保障，以突破国企改革为重点，力争打开改革开放的全新局面。

实现国企改革攻坚年决战决胜。按照“有利于职工就业、有利于产业发展、有利于对外开放、有利于社会稳定”的原则，一手抓人资分离，一手抓资产运营，集中精力在重点企业、重点问题上攻坚，初步完成市属国有经济布局的战略性调整。继续抓好人资分离，完成3万名职工劳动关系调整，职工身份置换率达到70%。大力实施“三个一批”战略，分类推进国企改制，市属国企改制面达到75%。完成铜厂、山针、玻璃瓶厂、三晋铝业等4户破产企业职工清算和补偿工作，其它10户计划内破产企业全部进入法律程序。对山西电机厂、太原工具厂、机床厂等企业进行搬迁改造，盘活现有土地和资产。构建国有土地开发平台，对条件具备的市属国企土地进行统一开发，设立国企改革专户，筹集和高效使用改制资金。构建债务处置运营平台，彻底处置在金融资产公司的30亿元不良债务，实现债务缩水解套。构建非主业资产管理服务平台，对非主业资产进行改制运营，促进主辅分离。构建再就业服务平台，通过有载体破产重组、小额贷款等方式，促进职工再就业。构建企业离退休人员管理服务平台，成立市属国有企业离休人员管理服务中心，加快推进国企退休人员社会化管理工作。

积极推进其它各项改革。在深化投融资体制改革上迈出步伐，重视发展和利用资本市场，大力吸引世行贷款和民间闲散资金。围绕民生与和谐社会建设，深化公共财政改革，调整优化支出结构，重点支持公共事业发展。加强政府采购监督管理，构建“阳光采购”。加快事业单位人事、分配、产权等各项制度改革，建立政事职责分开、单位自主用人、人员自主择业、政府依法管理、配套措施完善的分类管理体制。稳步推进市政公用事业改革。营造良好的金融生态环境，继续深化商行改革。加快户籍制度改革，实现城乡统一的户口登记制度。

提高对外贸易和交流合作水平。狠抓中博会、港洽会、沪洽会等签约项目的履约工作，密切跟踪、随时掌握意向项目的进展情况，切实提高外资实际到位率。调整出口商品结构，力求形成以机电产品、高新技术产品为龙头，资源性产品和农副产品为两翼的对外贸易新格局。引导有实力的企业开展省外、境外投资，鼓励企业参加各类经贸交流活动。全力配合省政府、商务部做好煤博会有关工作，举办好我省首个国家级展会。以俄罗斯“中国年”、中日建交35周年为契机，开展与俄罗斯瑟克特夫卡尔市、日本姬路市等国际友城的高层互访，拓宽交流领域，扩大交流成果。

（六）增强解决群众生产生活问题的能力，努力建设和谐太原

突出解决好人民群众最关心、最直接、最现实的就业、保障、教育、医疗、住房和安全等各类民生问题，加快推进基本公共服务均等化和社会公平进程。

千方百计扩大就业。着力抓好改制企业职工再就业，认真落实各项就业再就业扶持政策，有针对性地做好改制企业特别是新凯、铜厂等职工较多企业的再就业工作。认真研究解决大学生就业问题，挖掘用人单位信息，继续开展就业指导和岗前培训，提高大学生就业率。继续为零就业家庭提供公益性岗位。城镇新增就业岗位14万个，下岗失业再就业5.5万人，安置“4050”等就业困难人员9500人，城镇登记失业率控制在4%以内。

高起点推进新型社会保障体系建设。围绕社保事业跨越发展、创建中部地区社保先进城市的目标，创新社保模式，研究制定总体方案，高标准推进工作。制定出台《太原市社会保险管理条例》，把社会保险纳入法制化轨道。企业养老、失业、医疗、工伤保险参保人数分别达到63.5万人、73万人、93万人和38万人，重点把城市低保户和中、小学生纳入医疗保障范围，解决被征地农民和工矿塌陷区农民的养老保险问题，强力推进非公有制企业参保，强化保险征缴，实现应保尽保。加强住房公积金监管，确保住房公积金安全。完善城乡社会救助体系，支持慈善事业发展，保障困难群众生活。

大力发展社会事业。巩固提高学前三年教育和义务教育水平，继续实施“百校兴学”工程，促进义务教育的均衡发展。推进义务教育标准化建设，万柏林、尖草坪、晋源3个城区通过省达标验收。抓好农村中小学危房扫尾改造，彻底消除学校危房。大力发展职业教育，办好12所国家和省级重点职业学校，建设以数控技术应用等四大专业为主的国家级技能型紧缺人才培养培训基地，建设100个职业教育校外实习基地。提升高等学校办学水平，开工建设汾东教育城和太原大学新校区，优化整合省城高教资源。加快建设“和谐社区”。积极创建国家卫生城市，全面提高城乡卫生管理水平，大力发展社区卫生服务，增设50个社区卫生服务机构。巩固完善济困门诊、济困病房、专家医疗援助等措施，为困难群众尽可能提供优惠的医疗卫生服务。推进新型农村合作医疗，覆盖全市所有的行政村，参合率达到85%以上。培训150名乡镇卫生院卫技人员和800名乡村医生。加强人口和计划生育工作，稳定低生育水平，提高人口素质，人口出生率、自然增长率分别控制在8.3‰和4.3‰。大力开展全民健身活动，积极发展体育产业，开工建设滨河体育场，备战全国“六城会”和2008年奥运会，做好小轮车世锦赛暨奥运会预赛筹备工作。

高度重视安全生产和公共安全工作。健全安全生产监管体系，严格落实安全生产责任制，突出抓好煤矿安全生产，对私挖滥采行为始终保持高压态势，淘汰关闭40座矿井，全市矿井数量下降到200座以下。继续开展非煤矿山、危险化学品、民爆物品和建筑等重点领域的安全生产专项整治，做好道路交通安全和消防安全工作，加强对事故隐患和重大危险源的排查、监控和整改。加快城市供水、供气、集中供热等市政公用基础设施建设和应急基础设施、应急体系建设，提高应对各种公共突发事件的处置能力。建立健全农产品质量安全体系，提高农产品质量安全水平。完善食品药品监管体系，强化对重点品种、重点场所、重点区域的监控和管理。加强社会治安综合治理，开展基层安全和平安县区创建活动。深入推进严打整治斗争，依法严厉打击各类犯罪活动，为我市经济社会又好又快发展创造良好的社会治安环境。

坚持国防建设与经济建设协调发展。深入开展全民国防教育，扎实搞好双拥共建活动。切实维护国防和军事利益，维护军人和军属合法权益。加强民兵和预备役部队建设，进一步发挥在地方“三个文明”建设中的作用，积极为太原经济和社会发展作贡献。

档案、气象、地震、人防、地方志、国防后备力量建设、老龄、双拥、侨务、残疾人等各项工作，都要实现新发展，取得新成绩。

（七）围绕特色文化名城建设，推进文化产业和文化事业快速发展

致力文化创新，建设文化强市，充分发挥文化对经济社会进步的巨大驱动作用，在谋划和推进文化产业、文化事业发展、加强文化基础设施建设等方面取得新突破。

大力发展文化产业。制定、完善和实施各种有利于加快文化产业发展、激励文化创新的政策措施。加大资金投入，重点支持文化创新、精品生产和具有示范性、导向性的文化产业项目研发。围绕富士康文化产业园区、“华夏文明看山西”研究展示园区和民俗文化产业、民族艺术产业、历史文化产业板块建设，尽快把文化产业打造成我市新的支柱产业。深化文化体制改革，发挥市场在文化资源配置中的基础性作用，鼓励和吸引民间资本进入文化产业领域。

稳步发展文化事业。切实推进哲学社会科学研究、新闻出版、广播电视、文化艺术事业发展，加强物质、非物质文化遗产保护和风景名胜资源保护，强化文化市场管理。繁荣文艺精品创作，打造一批精品力作，提升我市文化影响力。繁荣群众文化，办好纪念傅山诞辰四百周年活动和第九届全国老年合唱节。广泛开展各类文化活动，营造高品位城市人文环境。积极抓好全国文化信息资源共享工程、农村电影放映“2131”工程和广播电视村村通工程。

加强文化基础设施建设。着眼于满足人民群众日益增长的精神文化需求，健全公共文化服务体系，提高市民的文化生活质量。筹建太原美术馆，突出抓好县、乡文化馆（站）、村文化活动室建设。

（八）加强社会主义民主法制和精神文明建设，为经济社会发展提供政治保障和精神动力

大力发展社会主义民主政治和先进文化，促进物质文明、政治文明和精神文明全面进步。

推进社会主义民主政治建设。各级政府要定期向人大及其常委会报告工作，自觉接受政协的民主监督，认真听取民主党派、工商联、无党派人士和各人民团体的意见，积极办理人大代表建议和政协提案，支持政协开展工作。要主动接受社会监督和舆论监督，虚心听取各方面意见，提高决策的科学化、民主化、规范化水平。充分发挥工会、共青团、妇联等人民团体的桥梁纽带作用。加强基层民主建设，努力扩大政务公开、村务公开和厂务公开的范围和内容，保障人民群众的选举权、知情权、参与权和监督权。

加快依法治市进程。认真贯彻“五五”普法规划和依法治市纲要，搞好宪法的学习宣传，着力提高公民宪法意识。推进“无假货市”创建活动，加强市场价格监管，深入整顿和规范市场经济秩序，严厉打击制假售假、商业欺诈等违法行为，查处一批有影响的大案要案。加强“信用太原”建设，推进商标战略实施。进一步整顿规范各类评比、检查活动，坚决制止乱收费、乱摊派、乱罚款、乱检查，切实减轻企业负担。落实民族宗教政策，依法管理民族宗教事务。

加强社会主义精神文明建设。强化理想信念教育，在全社会树立中国特色社会主义的共同理想，大力弘扬以爱国主义为核心的民族精神、以改革创新为核心的时代精神、以“争先、创新、奉献、和谐”为核心的太钢精神。深入开展以“八荣八耻”为主要内容的社会主义荣辱观教育，建立完善社会公德、职业道德、家庭美德和个人品德等思想道德体系，在全社会形成爱国守法、明礼诚信、团结友善、勤俭自强、敬业奉献的基本道德规范。发扬“兼容和谐、诚信卓越”的城市精神，深入开展精神文明创建活动，不断提高城乡文明程度，努力创建全国文明城市。

三、切实加强政府自身建设

各位代表！这次大会将选举产生新一届市政府领导班子，新一届政府肩负着光荣而艰巨的历史使命。坚持科学执政、民主执政、依法执政，树立充满活力和希望的政府形象，是太原344万人民的殷切期待，是率先科学发展的迫切要求。必须下大力气抓好政府自身建设，以良好的形象凝聚人心，以高效的工作鼓舞士气，以出色的政绩向全市人民交上一份满意的答卷。

（一）严格依法行政，建设法治政府

各级政府要切实提高依法行政水平，严格按照法定权限和程序行使职权、履行职责，做到有权必有责、用权受监督、违法受追究、侵权须赔偿。深化行政管理体制改革，严格机构编制管理。深入贯彻实施《全面推进依法行政实施纲要》，健全行政责任制、绩效考核制和对行政管理权限的监督机制。进一步明确行政执法权限，依法梳理各执法部门的“权力清单”，界定执法职责，明确执法主体，规范执法程序，坚决克服多头执法、执法不公等问题。加强行政效能监察，严格落实行政不作为问责制和过错责任追究制，用严密的制度、严格的管理、严明的纪律，提升政府约束力。今年，要以建章立制为基础，以推行行政执法责任制为主要内容，促进行政执法人员严格执法、公正执法、文明执法。

（二）加快职能转变，建设服务型政府

按照减少、简化、规范和监督审批的原则，继续深化行政审批制度改革，优化审批要素，整合审批权力，简化审批环节，明晰审批权限，下放审批权力，提高行政效率。加强电子政务建设，完善政府新闻发言人制度，实行重大决策事项公示，大力推行点题公开，积极完善政府便民热线。继续落实全程代办制、服务承诺制、首办负责制、限时办结制和超时默认制，对重大投资项目设立“绿色通道”，为群众和投资者提供优质的行政服务。今年，要以开展“环境创新攻坚年”活动为抓手，改进政府管理与服务方式，不断提高政府的执行力和公信力，真正创造“重商、亲商、安商、富商”的社会氛围。按照“扩网、提质、增效、便民”的要求，继续完善市、县（区）、乡（街道）、村（社区）四级政务服务体系，全部街道、社区和70%以上的乡镇、村都要建立政务服务机构，确保政务服务的“触角”向全市各个角落延伸。

（三）坚持廉洁从政，建设廉洁政府

认真贯彻党中央、国务院和省委、省政府关于反腐倡廉的各项规定，全面落实政府系统党风廉政建设责任制，建立健全惩治和预防腐败体系，逐步形成按制度办事、靠制度管人的反腐保廉长效机制。加强行政监察、审计监督和政府督查，坚决查处违纪违法案件，坚决惩处腐败分子，坚决纠正损害人民群众利益的不正之风，继续开展治理商业贿赂专项工作。加强公务员教育、管理和监督，建设勤政廉洁的公务员队伍。今年，要继续围绕群众反映强烈的突出问题，加大对卫生、教育、交通等领域不正之风的专项治理力度。

（四）切实转变作风，建设高效政府

各级政府、各部门和各级领导干部要全面落实省委《关于加强作风建设、狠抓落实的决定》精神，强化公仆观念，自觉把维护好、实现好、发展好最广大人民群众的根本利益作为制定政策、开展工作的出发点和落脚点，把全部心思放在为民发展上，把所有精力放在为民谋利上，把更多时间放在深入基层、调查研究、解决问题上。要紧紧围绕自身职责和工作目标，找准差距，自我加压，不服输、不畏难，比成绩、比奉献，在全市上下创造想干事、能干事、干成事的浓厚氛围，努力开创各项工作新局面。克服官僚主义和形式主义，切实精简会议、文件，减少应酬、接待，改进会风、文风，防止急功近利，力戒心浮气躁。以加强领导干部思想作风、学风、工作作风、领导作风、生活作风建设为重点，倡导和树立“八个方面”的良好风气。认真落实加强作风建设与廉政建设的22项制度，务求取得明显效果。

（五）突出绿色治理，建设节约环保政府

围绕政府绿色管理，制定绿色GDP核算体系、绿色考核标准以及绿色机关、绿色采购、绿色办公、绿色服务等标准。在建设资源节约型和环境友好型社会进程中，政府机关要率先垂范，实行绿色管理、绿色采购、绿色办公，坚决摒弃讲排场、比阔气的恶习，切实解决严重铺张浪费问题，树立节约光荣、浪费可耻的观念，从节约一度电、一滴水、一张纸做起，在节能、节水、节地、节材、“节会”、“节车”、“节吃”等方面立即行动起来，为全市做好表率，堵塞管理漏洞，努力降低行政成本。

各位代表，办好民生大事，是人民政府义不容辞的责任。今年要在改善群众生产、生活方面着力办好“十二件实事”：一是按照五年内基本完成城市棚户区改造任务的要求，启动城市棚户区改造试点工作。西山棚户区改造新开工建设住房35万平方米。采煤沉陷区治理新建住宅30万平方米，让5015户、1.7万人住进新居。二是建设安康居住工程住房91万平方米，9700余户困难居民搬进新居；开工建设廉租住房4500平方米；落实240户廉租住房保障对象。三是完成10个乡镇卫生院、100个村卫生所的改扩建，为30个乡镇卫生院配备基本的医疗设备，力争全市50%的乡村卫生机构达到省级标准。抓好农村“巡回医院”的扩建和高效运行，6支巡回医疗队每月下乡不少于20天，覆盖全市1013个行政村。严格执行全省药品集中招标采购价格，医院药品价格平均下降30%左右。四是改善农村学校办学条件，为64所农村寄宿制小学新建、改造暖气设施。完成807所农村中小学远程教育工程，使信息技术覆盖到每一所农村学校。五是关心帮助农村特困群众生活，新建12所农村县级敬老院、3所乡级敬老院，扩建、维修18所乡级敬老院，农村五保户全部实行按标施保。六是加强企业职工养老和工伤保险工作，提高企业退休人员基本养老金标准，基本实现所有煤矿、非煤矿山企业包括农民工在内的职工参加工伤保险。七是发展沼气1.5万户，解决10万农村人口饮水安全问题，新增村村通水泥（油）路22公里。八是实施农村地质灾害治理工程，对19个地质灾害村进行集中治理，让2000余户、6300余人住上安全住房。九是帮助农村就业和脱贫致富，分别完成引导性培训和阳光工程培训3.3万人、1.25万人，转移农村富余劳动力2万人，扶贫移民搬迁1450人，汾河水库移民搬迁7000人。十是深入推进“万村千乡市场工程”，改造建设农家店220个。十一是支持“三农”贷款100亿元，贷款扶持10万农户，扶持100个能够带动农民增收的龙头企业和20个农民专业合作社。十二是大力推进“蓝天碧水”工程和十大环保绿色创建活动，积极创建全国环保模范城，市区二级以上天数达到267天，二氧化硫、化学需氧量排放量分别控制在14万吨和3.06万吨以下。

各位代表！新的形势催人奋进，新的征程任重道远。新一届政府一定要在科学发展观的指引下，在省委、省政府和市委的坚强领导下，在市人大、市政协的监督支持下，以更加创新的精神、更加昂扬的斗志、更加务实的作风，锐意进取，奋发图强，为建设服务全省、影响全国、吸引世界的新太原而努力奋斗，以优异成绩迎接党的十七大胜利召开！
